# Poly[aqua­[μ-1,2-bis­(pyridin-4-yl)ethene-κ^2^ *N*:*N*′][μ-5-(diphenyl­phosphino­yl)iso­phthalato-κ^3^ *O* ^1^,*O* ^1′^:*O* ^3^]nickel(II)]

**DOI:** 10.1107/S1600536812031108

**Published:** 2012-07-14

**Authors:** Peng Zhang, Yu-Jie Liu, Kai-Hui Li, Guang-Rui Yang, Chong-Zhen Mei

**Affiliations:** a Institute of Environmental and Municipal Engineering, North China University of Water Conservancy and Electric Power, Zhengzhou 450011, People’s Republic of China; b School of Biochemical and Chemical Engineering, Nanyang Institute of Technology, Nanyang 473004, People’s Republic of China

## Abstract

In the title compound, [Ni(C_20_H_13_O_5_P)(C_12_H_10_N_2_)(H_2_O)]_*n*_, the Ni^II^ cation is coordinated by three O atoms from two 5-(diphenyl­phosphino­yl)isophthalate anions, two N atoms from two 1,2-bis­(pyridin-4-yl)ethene ligands and one water mol­ecule in a distorted octa­hedral geometry. Both 1,2-bis­(pyridin-4-yl)ethene and 5-(diphenyl­phosphino­yl)iso­phthal­ate bridge the Ni^II^ cations to form polymeric layers parallel to (001). In the crystal, O—H⋯O hydrogen bonding links layers into a three-dimensional supra­molecular structure.

## Related literature

For background to the network topologies and applications of coordination polymers, see: Maspoch *et al.* (2007 ▶); Ockwig *et al.* (2005 ▶); Zang *et al.* (2011 ▶). For a related structure, see: Desiraju (2004 ▶).
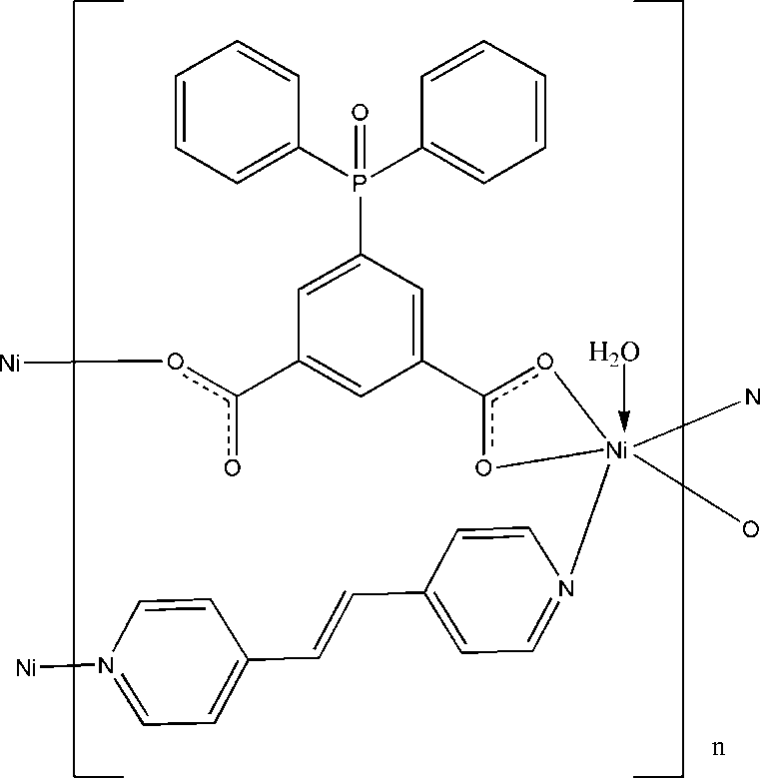


## Experimental

### 

#### Crystal data

[Ni(C_20_H_13_O_5_P)(C_12_H_10_N_2_)(H_2_O)]
*M* *_r_* = 623.22
Monoclinic, 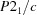

*a* = 10.1866 (3) Å
*b* = 13.6980 (3) Å
*c* = 21.7030 (6) Å
β = 111.174 (2)°
*V* = 2823.90 (13) Å^3^
*Z* = 4
Mo *K*α radiation
μ = 0.79 mm^−1^
*T* = 296 K
0.21 × 0.20 × 0.19 mm

#### Data collection

Bruker SMART APEXII CCD area-detector diffractometer
Absorption correction: multi-scan (*SADABS*; Bruker, 2001 ▶) *T*_min_ = 0.851, *T*_max_ = 0.864
11156 measured reflections
4971 independent reflections
3513 reflections with *I* > 2σ(*I*)
*R*_int_ = 0.043

#### Refinement

*R*[*F* ^2^ > 2σ(*F* ^2^)] = 0.047
*wR*(*F* ^2^) = 0.112
*S* = 0.99
4971 reflections
379 parameters
H-atom parameters constrained
Δρ_max_ = 0.66 e Å^−3^
Δρ_min_ = −0.33 e Å^−3^

### 

Data collection: *APEX2* (Bruker, 2007 ▶); cell refinement: *SAINT* (Bruker, 2007 ▶); data reduction: *SAINT*; program(s) used to solve structure: *SHELXTL* (Sheldrick, 2008 ▶); program(s) used to refine structure: *SHELXTL*; molecular graphics: *DIAMOND* (Brandenburg, 2010 ▶); software used to prepare material for publication: *SHELXTL*.

## Supplementary Material

Crystal structure: contains datablock(s) I, global. DOI: 10.1107/S1600536812031108/xu5570sup1.cif

Structure factors: contains datablock(s) I. DOI: 10.1107/S1600536812031108/xu5570Isup2.hkl

Additional supplementary materials:  crystallographic information; 3D view; checkCIF report

## Figures and Tables

**Table 1:** Selected bond lengths (Å)

Ni1—N1	2.145 (3)
Ni1—N2^i^	2.134 (3)
Ni1—O1	2.140 (2)
Ni1—O2	2.120 (2)
Ni1—O3^ii^	2.039 (2)
Ni1—O1*W*	2.046 (2)

Symmetry codes: (i) 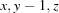; (ii) 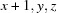.

**Table 2:** Hydrogen-bond geometry (Å, °)

*D*—H⋯*A*	*D*—H	H⋯*A*	*D*⋯*A*	*D*—H⋯*A*
O1*W*—H1*WA*⋯O5^iii^	0.85	1.84	2.684 (3)	173
O1*W*—H1*WB*⋯O4^ii^	0.85	1.81	2.622 (3)	158

Symmetry codes: (ii) 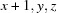; (iii) 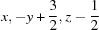.

**Fig. 1.**
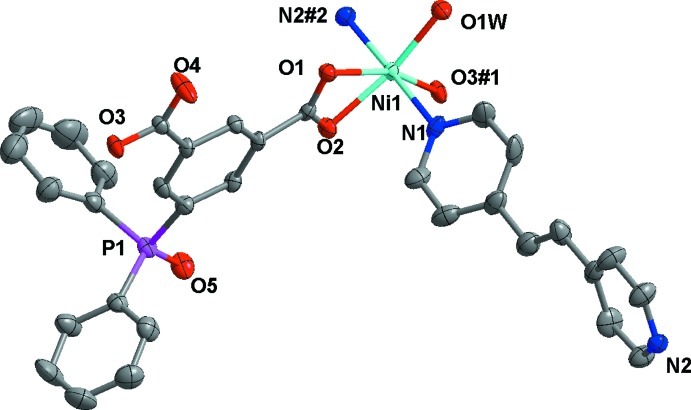
Metal coordination and atom labeling in title compound (thermal ellipsoids at 50% probability level). Irrespective hydrogen atoms are omitted for clarity. Symmetry codes: #1: x + 1, y, z; #2: x, y - 1, z.

**Fig. 2.**
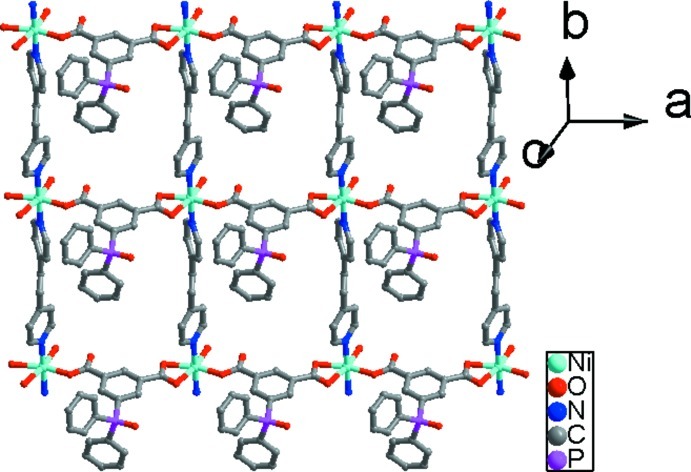
A view of the layer in compound 1

**Fig. 3.**
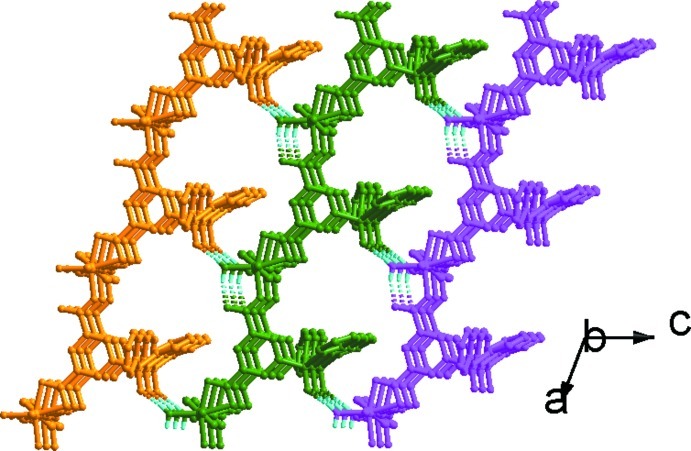
The three-dimensional supramolecular structure connected by hydrogen bonds. Dotted lines represent hydrogen bonds.

## supplementary crystallographic information

### Comment

Supramolecular coordination assemblies have received much attention due to their discovery of interesting topologies and crystal packing motifs, and the potential applications as functional materials (Maspoch *et al.*, 2007; Ockwig *et al.*, 2005). A great number of carboxylate-based ligands have been successfully employed in the generation of many novel structures (Zang *et al.*, 2011). To further explore various factors that influence the properties and construction of coordination compounds, we undertake synthetic and structural studies on one novel Ni(II) complex based on 5-(oxidediphenylphosphanyl)isophthalic acid (H2*L*) and 1,2-bis(pyridin-4-yl)ethene (bpe): [Ni(C_20_H_13_O_5_P)(C_12_H_10_N_2_)(H_2_O)]*_n_* (1).

X-ray crystallographic analysis revealed that the title compound crystallizes in monoclinic space group *P*2_1_/*c*. As shown in Fig. 1, the asymmetric unit consists of one Ni^II^ atom, one *L*^2-^ anion, one bpe ligand and one coordinated water molecule. Each metal center is six-coordinated by three O atoms from two *L*^2-^ anions, one O atom from the coordinated water molecule and two N atoms from different 1,2-bis(pyridin-4-yl)ethene ligands. Four atoms O1W, O1, O2 and O3#1 comprise the equatorial plane; while N1, N2#2 occupies the axial position.

Each *L*^2-^ ligand acts as a *µ*_2_-bridge linking two Ni^II^ atoms with one carboxylate group in monodentate fashion and the other one in chelating mode to form an infinite Ni- *L*^2-^ chain running along the *a*-axis. As depicted in Fig. 2, bpe ligand links adjacent chains running along the *b*-axis to form a (4,4)-layer with Ni1···Ni1 distance of 10.1866 (7) Å and 13.6980 (7) Å, respectively. Adjacent layers are associated together by O—H···O hydrogen bonds to achieve a three-dimensional supramolecular structure (Fig. 3). A further investigation reveals a more striking feature of title compound, *i.e.* two sets of symmetric related supamolecular structures are interlocked with each other to display a twofold interpenetrating architecture.

### Experimental

The title compound was synthesized hydrothermally in a Teflon-lined stainless teel container by heating a mixture of 5-(oxidediphenylphosphanyl)isophthalic acid (H2*L*) (0.0183 g, 0.05 mmol), 1,2-bis(4-pyridyl)ethane (bpe) (0.0091 g, 0.05 mmol), Ni(NO_3_)_2_.6H_2_O (0.0146 g, 0.05 mmol) and NaOH (0.0040 g, 0.1 mmol) in 7 ml of distilled water at 130°C for 3 days, and then cooled to room temperature. Green block crystals of 1 were obtained in 78% yield based on nickel.

### Refinement

H atoms were positioned geometrically and refined using a riding model, with C—H = 0.93 Å, *U*_iso_(H) = -1.2*U*_eq_(C) for aromatic H. The H atoms of the water molecules were located from the Fourier difference map and refined with suitable geometric restraints.

### Crystal data

**Table:**

[Ni(C_20_H_13_O_5_P)(C_12_H_10_N_2_)(H_2_O)]	*F*(000) = 1288
*M**_r_* = 623.22	*D*_x_ = 1.466 Mg m^−^^3^
Monoclinic, *P*2_1_/*c*	Mo *K*α radiation, λ = 0.71073 Å
Hall symbol: -P 2ybc	Cell parameters from 4354 reflections
*a* = 10.1866 (3) Å	θ = 3.0–29.2°
*b* = 13.6980 (3) Å	µ = 0.79 mm^−^^1^
*c* = 21.7030 (6) Å	*T* = 296 K
β = 111.174 (2)°	Block, green
*V* = 2823.90 (13) Å^3^	0.21 × 0.20 × 0.19 mm
*Z* = 4	

### Data collection

**Table:**

Bruker SMART APEXII CCD area-detector diffractometer	4971 independent reflections
Radiation source: fine-focus sealed tube	3513 reflections with *I* > 2σ(*I*)
Graphite monochromator	*R*_int_ = 0.043
ω scans	θ_max_ = 25.0°, θ_min_ = 3.0°
Absorption correction: multi-scan (*SADABS*; Bruker, 2001)	*h* = −12→11
*T*_min_ = 0.851, *T*_max_ = 0.864	*k* = −16→10
11156 measured reflections	*l* = −25→17

### Refinement

**Table:**

Refinement on *F*^2^	Primary atom site location: structure-invariant direct methods
Least-squares matrix: full	Secondary atom site location: difference Fourier map
*R*[*F*^2^ > 2σ(*F*^2^)] = 0.047	Hydrogen site location: inferred from neighbouring sites
*wR*(*F*^2^) = 0.112	H-atom parameters constrained
*S* = 0.99	*w* = 1/[σ^2^(*F*_o_^2^) + (0.0554*P*)^2^] where *P* = (*F*_o_^2^ + 2*F*_c_^2^)/3
4971 reflections	(Δ/σ)_max_ = 0.001
379 parameters	Δρ_max_ = 0.66 e Å^−^^3^
0 restraints	Δρ_min_ = −0.33 e Å^−^^3^

### Special details

**Table:**

Geometry. All e.s.d.'s (except the e.s.d. in the dihedral angle between two l.s. planes) are estimated using the full covariance matrix. The cell e.s.d.'s are taken into account individually in the estimation of e.s.d.'s in distances, angles and torsion angles; correlations between e.s.d.'s in cell parameters are only used when they are defined by crystal symmetry. An approximate (isotropic) treatment of cell e.s.d.'s is used for estimating e.s.d.'s involving l.s. planes.
Refinement. Refinement of *F*^2^ against ALL reflections. The weighted *R*-factor *wR* and goodness of fit *S* are based on *F*^2^, conventional *R*-factors *R* are based on *F*, with *F* set to zero for negative *F*^2^. The threshold expression of *F*^2^ > σ(*F*^2^) is used only for calculating *R*-factors(gt) *etc*. and is not relevant to the choice of reflections for refinement. *R*-factors based on *F*^2^ are statistically about twice as large as those based on *F*, and *R*- factors based on ALL data will be even larger.

### Fractional atomic coordinates and isotropic or equivalent isotropic displacement parameters (Å^2^)

**Table:**

	*x*	*y*	*z*	*U*_iso_*/*U*_eq_	
Ni1	0.42678 (4)	0.81298 (3)	0.86654 (2)	0.01907 (14)	
O1	0.2074 (2)	0.81261 (16)	0.85164 (11)	0.0224 (5)	
O2	0.3689 (2)	0.79683 (16)	0.95050 (11)	0.0248 (5)	
O3	−0.3597 (2)	0.81335 (18)	0.91252 (11)	0.0258 (6)	
O4	−0.2948 (2)	0.8308 (2)	0.82543 (12)	0.0509 (9)	
O5	0.2692 (2)	0.75667 (19)	1.15849 (12)	0.0362 (7)	
O1W	0.4295 (2)	0.82607 (17)	0.77310 (11)	0.0298 (6)	
H1WA	0.3729	0.8019	0.7373	0.045*	
H1WB	0.5171	0.8219	0.7804	0.045*	
N1	0.4236 (3)	0.9691 (2)	0.87333 (16)	0.0285 (7)	
N2	0.4261 (3)	1.65746 (19)	0.86079 (14)	0.0246 (7)	
C1	0.1303 (3)	0.8117 (2)	0.94387 (16)	0.0196 (7)	
C2	0.1668 (3)	0.7974 (2)	1.01139 (17)	0.0229 (8)	
H2	0.2610	0.7912	1.0383	0.027*	
C3	0.0630 (3)	0.7924 (2)	1.03897 (16)	0.0234 (8)	
C4	−0.0783 (3)	0.8035 (2)	0.99786 (16)	0.0232 (8)	
H4	−0.1478	0.8007	1.0162	0.028*	
C5	−0.1164 (3)	0.8184 (2)	0.93095 (16)	0.0202 (7)	
C6	−0.0103 (3)	0.8222 (2)	0.90399 (16)	0.0203 (7)	
H6	−0.0345	0.8318	0.8588	0.024*	
C7	0.2423 (3)	0.8062 (2)	0.91300 (17)	0.0202 (7)	
C8	−0.2691 (3)	0.8216 (2)	0.88494 (16)	0.0211 (7)	
C9	0.0171 (3)	0.8171 (3)	1.16355 (17)	0.0257 (8)	
C10	−0.0222 (4)	0.7750 (3)	1.2120 (2)	0.0422 (11)	
H10	−0.0102	0.7083	1.2199	0.051*	
C11	−0.0793 (5)	0.8311 (3)	1.2489 (2)	0.0564 (13)	
H11	−0.1076	0.8018	1.2808	0.068*	
C12	−0.0943 (5)	0.9305 (3)	1.2386 (2)	0.0484 (11)	
H12	−0.1304	0.9686	1.2643	0.058*	
C13	−0.0560 (4)	0.9727 (3)	1.1908 (2)	0.0439 (11)	
H13	−0.0673	1.0396	1.1835	0.053*	
C14	−0.0007 (4)	0.9168 (3)	1.15294 (19)	0.0363 (10)	
H14	0.0247	0.9463	1.1203	0.044*	
C15	0.0537 (4)	0.6256 (3)	1.11215 (18)	0.0323 (9)	
C16	−0.0846 (5)	0.5991 (3)	1.0956 (2)	0.0477 (11)	
H16	−0.1515	0.6454	1.0956	0.057*	
C17	−0.1247 (6)	0.5019 (4)	1.0788 (3)	0.0721 (16)	
H17	−0.2179	0.4829	1.0679	0.087*	
C18	−0.0238 (8)	0.4353 (4)	1.0787 (3)	0.0777 (18)	
H18	−0.0503	0.3712	1.0662	0.093*	
C19	0.1130 (7)	0.4611 (4)	1.0965 (3)	0.0748 (16)	
H19	0.1799	0.4144	1.0973	0.090*	
C20	0.1536 (5)	0.5558 (3)	1.1132 (2)	0.0511 (12)	
H20	0.2477	0.5734	1.1253	0.061*	
C21	0.3952 (4)	1.0150 (3)	0.92197 (19)	0.0360 (10)	
H21	0.3723	0.9770	0.9522	0.043*	
C22	0.3981 (4)	1.1145 (3)	0.9296 (2)	0.0395 (10)	
H22	0.3784	1.1421	0.9645	0.047*	
C23	0.4301 (4)	1.1727 (3)	0.8860 (2)	0.0350 (10)	
C24	0.4573 (4)	1.1272 (3)	0.8351 (2)	0.0494 (12)	
H24	0.4776	1.1641	0.8037	0.059*	
C25	0.4542 (4)	1.0257 (3)	0.8312 (2)	0.0450 (11)	
H25	0.4748	0.9963	0.7971	0.054*	
C26	0.4401 (4)	1.2818 (3)	0.8951 (2)	0.0409 (10)	
H26	0.4719	1.3061	0.9380	0.049*	
C27	0.4072 (4)	1.3439 (3)	0.8468 (2)	0.0384 (10)	
H27	0.3746	1.3186	0.8042	0.046*	
C28	0.4170 (4)	1.4519 (3)	0.8536 (2)	0.0328 (9)	
C29	0.5025 (4)	1.5007 (3)	0.9098 (2)	0.0410 (10)	
H29	0.5582	1.4656	0.9467	0.049*	
C30	0.5046 (4)	1.6028 (3)	0.91086 (19)	0.0368 (10)	
H30	0.5643	1.6339	0.9487	0.044*	
C31	0.3449 (4)	1.6099 (3)	0.8069 (2)	0.0397 (10)	
H31	0.2893	1.6466	0.7709	0.048*	
C32	0.3386 (4)	1.5088 (3)	0.8013 (2)	0.0437 (11)	
H32	0.2810	1.4798	0.7622	0.052*	
P1	0.11389 (9)	0.75019 (7)	1.12316 (4)	0.0244 (2)	

### Atomic displacement parameters (Å^2^)

**Table:**

	*U*^11^	*U*^22^	*U*^33^	*U*^12^	*U*^13^	*U*^23^
Ni1	0.0152 (2)	0.0192 (2)	0.0231 (3)	0.00013 (18)	0.00720 (18)	−0.0006 (2)
O1	0.0184 (12)	0.0279 (13)	0.0209 (13)	−0.0002 (10)	0.0070 (10)	−0.0014 (11)
O2	0.0141 (12)	0.0354 (14)	0.0253 (13)	0.0012 (10)	0.0076 (10)	−0.0010 (11)
O3	0.0135 (11)	0.0425 (15)	0.0223 (13)	−0.0006 (11)	0.0076 (10)	0.0043 (12)
O4	0.0190 (13)	0.112 (3)	0.0192 (15)	0.0003 (15)	0.0046 (11)	0.0110 (16)
O5	0.0215 (13)	0.0544 (18)	0.0269 (14)	0.0031 (12)	0.0018 (11)	0.0106 (13)
O1W	0.0211 (12)	0.0436 (16)	0.0222 (13)	−0.0066 (11)	0.0047 (10)	−0.0044 (12)
N1	0.0241 (16)	0.0178 (15)	0.0431 (19)	−0.0017 (13)	0.0116 (15)	−0.0006 (15)
N2	0.0257 (16)	0.0193 (15)	0.0289 (17)	0.0034 (13)	0.0099 (14)	−0.0002 (14)
C1	0.0152 (16)	0.0185 (17)	0.0263 (18)	0.0002 (14)	0.0089 (14)	0.0014 (16)
C2	0.0137 (16)	0.026 (2)	0.0261 (19)	−0.0001 (14)	0.0038 (14)	0.0024 (16)
C3	0.0181 (17)	0.028 (2)	0.0219 (18)	0.0004 (15)	0.0050 (15)	0.0003 (16)
C4	0.0175 (17)	0.031 (2)	0.0234 (19)	0.0032 (15)	0.0097 (15)	0.0038 (17)
C5	0.0149 (16)	0.0217 (18)	0.0243 (18)	0.0000 (14)	0.0072 (14)	0.0025 (16)
C6	0.0200 (17)	0.0232 (18)	0.0177 (17)	0.0009 (15)	0.0070 (14)	0.0030 (15)
C7	0.0160 (17)	0.0172 (17)	0.026 (2)	−0.0020 (14)	0.0060 (15)	−0.0016 (16)
C8	0.0192 (17)	0.0251 (19)	0.0194 (18)	0.0020 (15)	0.0074 (14)	0.0018 (16)
C9	0.0241 (18)	0.032 (2)	0.0191 (18)	0.0006 (17)	0.0051 (15)	0.0009 (17)
C10	0.065 (3)	0.032 (2)	0.036 (2)	0.007 (2)	0.026 (2)	0.004 (2)
C11	0.084 (4)	0.061 (3)	0.043 (3)	0.014 (3)	0.045 (3)	0.009 (2)
C12	0.059 (3)	0.052 (3)	0.038 (2)	0.016 (2)	0.021 (2)	−0.005 (2)
C13	0.049 (3)	0.033 (2)	0.045 (3)	0.008 (2)	0.013 (2)	0.000 (2)
C14	0.044 (2)	0.034 (2)	0.032 (2)	0.0027 (19)	0.0146 (19)	0.0090 (19)
C15	0.044 (2)	0.030 (2)	0.027 (2)	0.0013 (19)	0.0182 (19)	0.0034 (18)
C16	0.053 (3)	0.037 (3)	0.051 (3)	−0.007 (2)	0.017 (2)	−0.004 (2)
C17	0.084 (4)	0.066 (4)	0.064 (4)	−0.031 (3)	0.023 (3)	−0.004 (3)
C18	0.131 (6)	0.032 (3)	0.070 (4)	−0.015 (4)	0.037 (4)	−0.005 (3)
C19	0.113 (5)	0.039 (3)	0.080 (4)	0.018 (3)	0.044 (4)	0.004 (3)
C20	0.067 (3)	0.038 (3)	0.058 (3)	0.009 (2)	0.035 (3)	0.008 (2)
C21	0.041 (2)	0.029 (2)	0.030 (2)	−0.0027 (18)	0.0025 (19)	−0.0016 (18)
C22	0.038 (2)	0.029 (2)	0.043 (3)	0.0035 (19)	0.005 (2)	−0.009 (2)
C23	0.029 (2)	0.026 (2)	0.052 (3)	−0.0003 (17)	0.0153 (19)	−0.005 (2)
C24	0.060 (3)	0.025 (2)	0.079 (4)	−0.009 (2)	0.044 (3)	0.009 (2)
C25	0.055 (3)	0.025 (2)	0.070 (3)	0.001 (2)	0.040 (3)	−0.004 (2)
C26	0.041 (2)	0.034 (2)	0.048 (3)	−0.0032 (19)	0.017 (2)	−0.005 (2)
C27	0.046 (2)	0.034 (2)	0.043 (3)	0.0019 (19)	0.025 (2)	0.007 (2)
C28	0.032 (2)	0.0233 (19)	0.050 (3)	−0.0040 (17)	0.022 (2)	−0.005 (2)
C29	0.049 (3)	0.025 (2)	0.045 (3)	0.0120 (19)	0.013 (2)	0.017 (2)
C30	0.040 (2)	0.031 (2)	0.032 (2)	−0.0011 (19)	0.0034 (19)	0.0004 (19)
C31	0.040 (2)	0.027 (2)	0.042 (3)	0.0008 (19)	0.003 (2)	0.001 (2)
C32	0.049 (3)	0.027 (2)	0.045 (3)	−0.004 (2)	0.005 (2)	−0.005 (2)
P1	0.0209 (5)	0.0307 (5)	0.0206 (5)	0.0027 (4)	0.0062 (4)	0.0039 (4)

### Geometric parameters (Å, º)

**Table:**

Ni1—N1	2.145 (3)	C12—C13	1.363 (6)
Ni1—N2^i^	2.134 (3)	C12—H12	0.9300
Ni1—O1	2.140 (2)	C13—C14	1.383 (5)
Ni1—O2	2.120 (2)	C13—H13	0.9300
Ni1—O3^ii^	2.039 (2)	C14—H14	0.9300
Ni1—O1W	2.046 (2)	C15—C16	1.372 (5)
O1—C7	1.251 (4)	C15—C20	1.390 (5)
O2—C7	1.259 (4)	C15—P1	1.799 (4)
O3—C8	1.273 (4)	C16—C17	1.401 (6)
O3—Ni1^iii^	2.039 (2)	C16—H16	0.9300
O4—C8	1.228 (4)	C17—C18	1.375 (7)
O5—P1	1.491 (2)	C17—H17	0.9300
O1W—H1WA	0.8500	C18—C19	1.352 (7)
O1W—H1WB	0.8500	C18—H18	0.9300
N1—C25	1.320 (5)	C19—C20	1.371 (6)
N1—C21	1.347 (5)	C19—H19	0.9300
N2—C30	1.324 (4)	C20—H20	0.9300
N2—C31	1.333 (5)	C21—C22	1.371 (5)
N2—Ni1^iv^	2.134 (3)	C21—H21	0.9300
C1—C6	1.387 (4)	C22—C23	1.366 (5)
C1—C2	1.389 (5)	C22—H22	0.9300
C1—C7	1.520 (4)	C23—C24	1.381 (6)
C2—C3	1.392 (4)	C23—C26	1.505 (5)
C2—H2	0.9300	C24—C25	1.393 (5)
C3—C4	1.401 (4)	C24—H24	0.9300
C3—P1	1.805 (3)	C25—H25	0.9300
C4—C5	1.376 (5)	C26—C27	1.296 (5)
C4—H4	0.9300	C26—H26	0.9300
C5—C6	1.403 (4)	C27—C28	1.487 (5)
C5—C8	1.516 (4)	C27—H27	0.9300
C6—H6	0.9300	C28—C32	1.370 (5)
C9—C10	1.380 (5)	C28—C29	1.388 (5)
C9—C14	1.387 (5)	C29—C30	1.399 (5)
C9—P1	1.790 (3)	C29—H29	0.9300
C10—C11	1.380 (5)	C30—H30	0.9300
C10—H10	0.9300	C31—C32	1.389 (5)
C11—C12	1.380 (6)	C31—H31	0.9300
C11—H11	0.9300	C32—H32	0.9300
			
O3^ii^—Ni1—O1W	95.22 (9)	C14—C13—H13	119.8
O3^ii^—Ni1—O2	99.11 (9)	C13—C14—C9	120.2 (4)
O1W—Ni1—O2	165.63 (9)	C13—C14—H14	119.9
O3^ii^—Ni1—N2^i^	90.65 (10)	C9—C14—H14	119.9
O1W—Ni1—N2^i^	91.73 (10)	C16—C15—C20	119.9 (4)
O2—Ni1—N2^i^	87.14 (10)	C16—C15—P1	123.7 (3)
O3^ii^—Ni1—O1	160.95 (9)	C20—C15—P1	116.0 (3)
O1W—Ni1—O1	103.75 (9)	C15—C16—C17	119.7 (5)
O2—Ni1—O1	61.96 (8)	C15—C16—H16	120.2
N2^i^—Ni1—O1	90.45 (10)	C17—C16—H16	120.2
O3^ii^—Ni1—N1	90.23 (10)	C18—C17—C16	118.9 (5)
O1W—Ni1—N1	89.24 (11)	C18—C17—H17	120.5
O2—Ni1—N1	91.67 (10)	C16—C17—H17	120.5
N2^i^—Ni1—N1	178.62 (11)	C19—C18—C17	121.3 (5)
O1—Ni1—N1	88.38 (10)	C19—C18—H18	119.3
C7—O1—Ni1	87.68 (18)	C17—C18—H18	119.3
C7—O2—Ni1	88.37 (19)	C18—C19—C20	120.3 (5)
C8—O3—Ni1^iii^	126.6 (2)	C18—C19—H19	119.9
Ni1—O1W—H1WA	128.7	C20—C19—H19	119.9
Ni1—O1W—H1WB	101.5	C19—C20—C15	119.9 (5)
H1WA—O1W—H1WB	117.6	C19—C20—H20	120.1
C25—N1—C21	116.1 (3)	C15—C20—H20	120.1
C25—N1—Ni1	121.6 (3)	N1—C21—C22	123.9 (4)
C21—N1—Ni1	122.2 (2)	N1—C21—H21	118.0
C30—N2—C31	116.3 (3)	C22—C21—H21	118.0
C30—N2—Ni1^iv^	121.9 (2)	C23—C22—C21	119.8 (4)
C31—N2—Ni1^iv^	121.8 (2)	C23—C22—H22	120.1
C6—C1—C2	119.7 (3)	C21—C22—H22	120.1
C6—C1—C7	120.0 (3)	C22—C23—C24	117.3 (3)
C2—C1—C7	120.0 (3)	C22—C23—C26	120.7 (4)
C1—C2—C3	120.3 (3)	C24—C23—C26	122.0 (4)
C1—C2—H2	119.8	C23—C24—C25	119.5 (4)
C3—C2—H2	119.8	C23—C24—H24	120.2
C2—C3—C4	119.1 (3)	C25—C24—H24	120.2
C2—C3—P1	117.8 (2)	N1—C25—C24	123.3 (4)
C4—C3—P1	122.2 (3)	N1—C25—H25	118.3
C5—C4—C3	121.4 (3)	C24—C25—H25	118.3
C5—C4—H4	119.3	C27—C26—C23	124.0 (4)
C3—C4—H4	119.3	C27—C26—H26	118.0
C4—C5—C6	118.6 (3)	C23—C26—H26	118.0
C4—C5—C8	122.1 (3)	C26—C27—C28	125.8 (4)
C6—C5—C8	119.1 (3)	C26—C27—H27	117.1
C1—C6—C5	120.9 (3)	C28—C27—H27	117.1
C1—C6—H6	119.6	C32—C28—C29	116.5 (3)
C5—C6—H6	119.6	C32—C28—C27	119.1 (4)
O1—C7—O2	121.8 (3)	C29—C28—C27	124.3 (4)
O1—C7—C1	119.7 (3)	C28—C29—C30	119.7 (4)
O2—C7—C1	118.5 (3)	C28—C29—H29	120.1
O4—C8—O3	126.0 (3)	C30—C29—H29	120.1
O4—C8—C5	118.3 (3)	N2—C30—C29	123.5 (4)
O3—C8—C5	115.7 (3)	N2—C30—H30	118.3
C10—C9—C14	118.8 (3)	C29—C30—H30	118.3
C10—C9—P1	121.5 (3)	N2—C31—C32	123.9 (4)
C14—C9—P1	119.0 (3)	N2—C31—H31	118.0
C11—C10—C9	120.7 (4)	C32—C31—H31	118.0
C11—C10—H10	119.7	C28—C32—C31	120.0 (4)
C9—C10—H10	119.7	C28—C32—H32	120.0
C12—C11—C10	120.0 (4)	C31—C32—H32	120.0
C12—C11—H11	120.0	O5—P1—C9	112.80 (16)
C10—C11—H11	120.0	O5—P1—C15	111.85 (16)
C13—C12—C11	119.8 (4)	C9—P1—C15	109.22 (16)
C13—C12—H12	120.1	O5—P1—C3	111.41 (15)
C11—C12—H12	120.1	C9—P1—C3	108.90 (16)
C12—C13—C14	120.5 (4)	C15—P1—C3	102.11 (16)
C12—C13—H13	119.8		
			
O3^ii^—Ni1—O1—C7	−4.1 (4)	C20—C15—C16—C17	−1.1 (6)
O1W—Ni1—O1—C7	−178.89 (19)	P1—C15—C16—C17	170.9 (4)
O2—Ni1—O1—C7	2.69 (18)	C15—C16—C17—C18	−0.6 (7)
N2^i^—Ni1—O1—C7	89.2 (2)	C16—C17—C18—C19	2.1 (8)
N1—Ni1—O1—C7	−90.1 (2)	C17—C18—C19—C20	−1.9 (9)
O3^ii^—Ni1—O2—C7	175.10 (19)	C18—C19—C20—C15	0.2 (8)
O1W—Ni1—O2—C7	−8.9 (5)	C16—C15—C20—C19	1.3 (7)
N2^i^—Ni1—O2—C7	−94.70 (19)	P1—C15—C20—C19	−171.3 (4)
O1—Ni1—O2—C7	−2.67 (18)	C25—N1—C21—C22	−0.6 (5)
N1—Ni1—O2—C7	84.60 (19)	Ni1—N1—C21—C22	177.1 (3)
O3^ii^—Ni1—N1—C25	80.4 (3)	N1—C21—C22—C23	0.6 (6)
O1W—Ni1—N1—C25	−14.8 (3)	C21—C22—C23—C24	0.5 (6)
O2—Ni1—N1—C25	179.5 (3)	C21—C22—C23—C26	−177.3 (3)
O1—Ni1—N1—C25	−118.6 (3)	C22—C23—C24—C25	−1.4 (6)
O3^ii^—Ni1—N1—C21	−97.2 (3)	C26—C23—C24—C25	176.4 (4)
O1W—Ni1—N1—C21	167.6 (3)	C21—N1—C25—C24	−0.3 (6)
O2—Ni1—N1—C21	1.9 (3)	Ni1—N1—C25—C24	−178.1 (3)
O1—Ni1—N1—C21	63.8 (3)	C23—C24—C25—N1	1.3 (7)
C6—C1—C2—C3	0.7 (5)	C22—C23—C26—C27	−147.7 (4)
C7—C1—C2—C3	−173.4 (3)	C24—C23—C26—C27	34.7 (6)
C1—C2—C3—C4	−0.9 (5)	C23—C26—C27—C28	−179.3 (3)
C1—C2—C3—P1	168.1 (3)	C26—C27—C28—C32	−158.9 (4)
C2—C3—C4—C5	0.5 (5)	C26—C27—C28—C29	21.8 (6)
P1—C3—C4—C5	−168.0 (3)	C32—C28—C29—C30	0.2 (6)
C3—C4—C5—C6	0.2 (5)	C27—C28—C29—C30	179.6 (3)
C3—C4—C5—C8	174.6 (3)	C31—N2—C30—C29	−1.7 (6)
C2—C1—C6—C5	0.0 (5)	Ni1^iv^—N2—C30—C29	177.3 (3)
C7—C1—C6—C5	174.1 (3)	C28—C29—C30—N2	1.4 (6)
C4—C5—C6—C1	−0.4 (5)	C30—N2—C31—C32	0.5 (6)
C8—C5—C6—C1	−175.0 (3)	Ni1^iv^—N2—C31—C32	−178.5 (3)
Ni1—O1—C7—O2	−4.7 (3)	C29—C28—C32—C31	−1.3 (6)
Ni1—O1—C7—C1	174.1 (3)	C27—C28—C32—C31	179.3 (3)
Ni1—O2—C7—O1	4.7 (3)	N2—C31—C32—C28	1.1 (7)
Ni1—O2—C7—C1	−174.1 (3)	C10—C9—P1—O5	−88.3 (3)
C6—C1—C7—O1	1.1 (5)	C14—C9—P1—O5	82.1 (3)
C2—C1—C7—O1	175.2 (3)	C10—C9—P1—C15	36.7 (4)
C6—C1—C7—O2	179.9 (3)	C14—C9—P1—C15	−152.9 (3)
C2—C1—C7—O2	−6.0 (5)	C10—C9—P1—C3	147.5 (3)
Ni1^iii^—O3—C8—O4	−0.1 (5)	C14—C9—P1—C3	−42.1 (3)
Ni1^iii^—O3—C8—C5	−179.3 (2)	C16—C15—P1—O5	164.2 (3)
C4—C5—C8—O4	−176.7 (3)	C20—C15—P1—O5	−23.6 (4)
C6—C5—C8—O4	−2.4 (5)	C16—C15—P1—C9	38.6 (4)
C4—C5—C8—O3	2.5 (5)	C20—C15—P1—C9	−149.2 (3)
C6—C5—C8—O3	176.9 (3)	C16—C15—P1—C3	−76.6 (4)
C14—C9—C10—C11	0.5 (6)	C20—C15—P1—C3	95.6 (3)
P1—C9—C10—C11	171.0 (3)	C2—C3—P1—O5	18.4 (3)
C9—C10—C11—C12	−1.6 (7)	C4—C3—P1—O5	−172.9 (3)
C10—C11—C12—C13	1.7 (7)	C2—C3—P1—C9	143.5 (3)
C11—C12—C13—C14	−0.8 (7)	C4—C3—P1—C9	−47.9 (3)
C12—C13—C14—C9	−0.3 (6)	C2—C3—P1—C15	−101.1 (3)
C10—C9—C14—C13	0.4 (6)	C4—C3—P1—C15	67.5 (3)
P1—C9—C14—C13	−170.3 (3)		

Symmetry codes: (i) *x*, *y*−1, *z*; (ii) *x*+1, *y*, *z*; (iii) *x*−1, *y*, *z*; (iv) *x*, *y*+1, *z*.

### Hydrogen-bond geometry (Å, º)

**Table:**

*D*—H···*A*	*D*—H	H···*A*	*D*···*A*	*D*—H···*A*
O1*W*—H1*WA*···O5^v^	0.85	1.84	2.684 (3)	173
O1*W*—H1*WB*···O4^ii^	0.85	1.81	2.622 (3)	158

Symmetry codes: (ii) *x*+1, *y*, *z*; (v) *x*, −*y*+3/2, *z*−1/2.
